# Phosphate Groups in the Lipid A Moiety Determine the Effects of LPS on Hepatic Stellate Cells: A Role for LPS-Dephosphorylating Activity in Liver Fibrosis

**DOI:** 10.3390/cells9122708

**Published:** 2020-12-17

**Authors:** Marlies Schippers, Eduard Post, Ilse Eichhorn, Jitske Langeland, Leonie Beljaars, Madhu S. Malo, Richard A. Hodin, José Luis Millán, Yury Popov, Detlef Schuppan, Klaas Poelstra

**Affiliations:** 1 Department of Nanomedice and Drug Targeting, Groningen Research Institute of Pharmacy (GRIP), University of Groningen, Antonius Deusinglaan 1, 9713 AV Groningen, The Netherlands; a.m.t.schippers@gmail.com (M.S.); e.post@rug.nl (E.P.); i.a.eichhorn@rug.nl (I.E.); jitskelangeland@gmail.com (J.L.); e.beljaars@rug.nl (L.B.); 2 Department of Surgery, Massachusetts General Hospital, Harvard Medical School, Boston, MA 02114, USA; madhumalo@hotmail.com (M.S.M.); rhodin@mgh.harvard.edu (R.A.H.); 3 Bangladesh Institute of Research and Rehabilitation for Diabetes, Endocrine and Metabolic Disorders (BIRDEM), Dhaka 1000, Bangladesh; 4 Sanford Burnham Prebys Medical Discovery Institute, La Jolla, CA 92037, USA; millan@sbpdiscovery.org; 5 Beth Israel Deaconess Medical Center, Harvard Medical School, Boston, MA 02215, USA; ypopov@bidmc.harvard.edu (Y.P.); detlef.schuppan@unimedizin-mainz.de (D.S.); 6 Medical Center of the Johannes Gutenberg University of Mainz, 55131 Mainz, Germany

**Keywords:** lipopolysaccharide, lipid A, hepatic stellate cells, liver fibrosis, alkaline phosphatase

## Abstract

Alkaline phosphatase (AP) activity is highly upregulated in plasma during liver diseases. Previously, we demonstrated that AP is able to detoxify lipopolysaccharide (LPS) by dephosphorylating its lipid A moiety. Because a role of gut-derived LPS in liver fibrogenesis has become evident, we now examined the relevance of phosphate groups in the lipid A moiety in this process. The effects of mono-phosphoryl and di-phosphoryl lipid A (MPLA and DPLA, respectively) were studied in vitro and LPS-dephosphorylating activity was studied in normal and fibrotic mouse and human livers. The effects of intestinal AP were studied in mice with CCL4-induced liver fibrosis. DPLA strongly stimulated fibrogenic and inflammatory activities in primary rat hepatic stellate cells (rHSCs) and RAW264.7 macrophages with similar potency as full length LPS. However, MPLA did not affect any of the parameters. LPS-dephosphorylating activity was found in mouse and human livers and was strongly increased during fibrogenesis. Treatment of fibrotic mice with intravenous intestinal-AP significantly attenuated intrahepatic desmin^+^− and αSMA^+^ −HSC and CD68^+^− macrophage accumulation. In conclusion, the lack of biological activity of MPLA, contrasting with the profound activities of DPLA, shows the relevance of LPS-dephosphorylating activity. The upregulation of LPS-dephosphorylating activity in fibrotic livers and the protective effects of exogenous AP during fibrogenesis indicate an important physiological role of intestinal-derived AP during liver fibrosis.

## 1. Introduction

One of the serum factors that changes profoundly during hepatic fibrosis, as well as in many other liver diseases, is serum alkaline phosphatase (AP) activity. In fact, this serum enzyme is routinely used as a marker for liver diseases. We were the first to show that AP is able to dephosphorylate lipopolysaccharide (LPS) at physiological pH levels [1,2]. Most biological effects of LPS are due to the presence of the lipid A moiety [3], which is a fairly conserved part of this otherwise highly variable molecule among Gram-negative bacterial species. Phosphate groups in this lipid A region critically determine the biological effects of LPS [3]; they affect the binding of LPS to TLR4 and subsequent NFĸB activation [4], and hence the host response towards LPS molecules [5,6] and even mediate resistance to antimicrobial peptides [7]. 

We previously showed that AP is able to detoxify LPS by removal of at least one of these phosphate groups [1,2,8,9]. Since then, many studies on the effects of AP in several LPS-mediated diseases have been performed [10,11] and clinical phase IIa trials with exogenous AP in patients with LPS-associated pathologies, such as inflammatory bowel diseases and acute renal failure, have been successfully performed [12,13,14]. More clinical trials, up to phase III, are in progress now. The results of the STOP-Aki Phase II trial [15] in 301 patients with acute kidney injury have recently been published [16,17], showing a beneficial effect of AP treatment on kidney function 3 to 4 weeks after the start of treatment and a significant beneficial effect on all-cause mortality at day 28 in patients with acute kidney injury [17]. To examine whether this enzyme can also be applied as a therapeutic entity during hepatic fibrosis, which is characterised by increased LPS exposure, its physiological role in pathological conditions needs to be unravelled.

Gut-derived LPS is an important pathogenic factor in alcohol-induced hepatitis (ASH), in non-alcoholic steato-hepatitis (NASH), and in several other liver diseases [18,19,20,21,22,23,24,25]. During these liver diseases, translocation of bacterial products into the portal vein leads to enhanced LPS exposure of hepatic cells [25,26,27,28,29]. Within the liver, all resident cells are able to respond to LPS. Hepatocytes, Kupffer cells, endothelial cells, bile duct epithelial cells, and hepatic stellate cells (HSC) are all known to express CD14 and Toll-like receptor-4 (TLR4) [18,25,30], and the expression of these receptors on many of these cells is upregulated during fibrogenesis [29]. So, concomitant with an increased intestinal translocation of LPS, the expression of LPS-responsive receptors increases [26,31]. Recent studies have shown that AP-treatment protects mice from alcohol-induced liver disease [32] and rats with acute-on-chronic liver failure [33].

To examine the role of intestinal AP in hepatic cells, we thus studied the direct effects of wild-type LPS as well as phosphorylated and de-phosphorylated lipid A on primary HSC. We also studied LPS-dephosphorylating activity in normal and fibrotic livers, and studied the effect of exogenously administered intestinal AP on liver fibrogenesis in vivo using the CCl_4_ model, which is characterized by increased translocation of LPS from the intestinal lumen into the portal vein [34,35,36]. Our studies reveal an important structure-activity relationship between LPS and its profibrogenic effect. The significance of phosphate groups in the lipid A moiety illustrates the relevance of lipid A dephosphorylating activity. This activity is upregulated in the liver during fibrogenesis. Our study adds new insights to the organization of the liver–gut axis during liver fibrosis.

## 2. Materials and Methods

### 2.1. Materials

The following primary antibodies were used: goat anti-collagen I (Southern Biotechnology Associates, Birmingham, AL, USA), monoclonal rat anti-mouse CD68 (AbD Serotec, Düsseldorf, Germany), goat anti-Desmin (Santa Cruz Biotechnology, Heidelberg, Germany), goat anti-chitinase 3-like/ECF-L (YM-1), rabbit anti-IRF5 (Protein Tech, Manchester, UK), and APC/Cy7-conjugated anti-MHCII antibody (Biolegend, San Diego, CA, USA). Species-specific horseradish peroxidase-conjugated secondary antibodies were purchased from Dako Denmark A/S (Glostrup, Denmark).

LPS from *Escherichia coli* serotype O55:B5 was obtained from Sigma Chemical Co (St. Louis, MI, USA). Mono-phosphoryl lipid A (MPLA) and di-phosphoryl lipid A (DPLA), both derived from *Salmonella minnesota* R595, were purchased from List Biological Laboratories (Campbell, CA, USA, product #401 and product #304, respectively). Calf-intestinal alkaline phosphatase (5000 mUnits/mL) was a gift from AM-Pharma BV, the Netherlands.

### 2.2. In Vitro Studies: Effects of Mono- and Di-Phosphoryl Lipid A on Macrophages and Hepatic Stellate Cells

We examined the effects of wild-type LPS (which served as positive control), di-phosphoryl lipid A, and mono-phosphoryl lipid A on mouse RAW264.7 macrophages (ECACC, Porton Down, Wiltshire, UK) and in cultures of primary rat hepatic stellate cells (rHSCs), isolated from normal rat livers. rHSC (200,000 cells/mL) or RAW cells (100,000 cells/mL) were incubated for 24 h with wild-type LPS from *E. coli* (100 ng/mL) or DPLA (100 ng/mL) or MPLA (100 ng/mL), both from *S. minnesota* R595. RAW cells were analyzed for % live cells and Major Histocompatibility Complex (MHC) class II expression using FACS analysis as previously described [37], and analyzed using standard rt-PCR methods with SybrGreen according to the manufacturer’s instructions (GC Biotech). The primers used in this study are presented in Table 1. β-Actin was used as house-keeping gene.

### 2.3. NO Assay

The production of NO was measured in supernatants of stimulated RAW 264.7 cells, as described earlier [9]. Briefly, cells were seeded in a 96-well plate at a density of 1 × 10^5^ cells and grown overnight. Subsequently, media were replaced and either DMEM including 5% fetal calf serum (=vehicle) or DMEM with 5% fetal calf serum supplemented with LPS, DPLA, or MPLA (all in concentration of 100 ng/mL) was added and cells were incubated for 24 h. After incubation, 100 µL of the supernatants was measured by adding 100 µL Griess reagents (200 mg sulphanilamide + 0.5 mL phosphoric acid + 20 mg N-naphthyl ethylene diamine + 19.5 mL distilled water). The absorbance was measured at 550 nm in a microplate ELISA reader. NaNO_2_ diluted in DMEM was used to create a standard curve.

### 2.4. Animal Experiments

All experiments with animals were approved by the Animal Ethics Committee of the University of Groningen, the Netherlands. All animals were purchased from Harlan (Zeist, The Netherlands).

CCl_4_-induced advanced liver fibrosis model: Male Balb/c mice (20–22 g) were injected intraperitoneally (i.p.) twice a week with increasing doses of CCl_4_ diluted in olive oil (week 1, 0.5 mL/kg; week 2, 0.8 mL/kg; and week 3–8, 1 mL/kg prepared in olive oil). Control mice (*n* = 6) received no CCl_4_ nor oil. At week 7 and 8, mice were treated with intestinal alkaline phosphatase (iAP: 500 mUnits /mouse, i.v. three times a week (*n* = 5) or saline (*n* = 6). All mice were sacrificed at week 8. To assess the acute effects of CCl4 on hepatic and serum AP activity, an additional group of mice (*n* = 6) was sacrificed 24 h after the first i.p.injection of CCl_4_.

### 2.5. Human Tissue Samples

The use of human tissue was approved by the Medical Ethical Committee of the University Medical Center Groningen (UMCG), according to the Dutch legislation and the Code of Conduct for dealing responsibly with human tissue in the context of health research (www.federa.org), foregoing the need for written consent for “further use” of anonymous human tissue. Normal human liver tissue was obtained from donor livers discarded for transplantation for technical reasons. Cirrhotic human liver tissue was obtained from patients undergoing liver transplantation; indications for transplantation were primary sclerosing cholangitis (PSC), primary biliary cirrhosis (PBC), congenital cirrhosis, alcohol-induced liver disease, acute liver failure, and Wilson’s cirrhosis. All human liver material was anonymized prior to storage, and known patient characteristics are listed in Supplementary Table S1.

### 2.6. Immunohistochemistry, Enzyme Histochemistry and Quantitative Analysis of Sections

Collagen I, Desmin, and CD68 expression was stained in livers of CCL4-treated animals using cryostat sections (4 μm) according to standard indirect immunoperoxidase methods. Stainings were visualized using 3-amino-9-ethylcarbazole or NovaRed (Vector Laboratories) and quantified by analysing complete sections from three different liver lobes of each animal at a magnification of 10 × 10 using the Cell D image analysing software (Olympus, Hamburg, Germany).

#### 2.6.1. Alkaline Phosphatase Staining 

0.1% Fast Blue and 0.02% Naphthol AS-MX-phosphate were dissolved in 0.1 M TRIS/HCl pH 8.2 buffer containing 2 mM MgCl_2_. The solution was filtered and incubated on cryo-sections for 30 min at 37 °C. Sections were washed in Phosphate-buffered saline (PBS) and nuclei were counterstained with Mayer’s hematoxylin.

#### 2.6.2. Histochemical Detection of LPS-Dephosphorylation 

LPS-dephosphorylating activity in the livers of mice and human tissue specimen was examined by incubating 4 μm cryostat sections with LPS (from *Escherichia coli* (serotype O55:B5, Sigma St. Louis, MO, USA)) as substrate in a final concentration of 0.5 mg/mL, as described before [38]. Briefly, sections were fixed in 4% formalin-macrodex and subsequently incubated for 120 min in Tris-HCl buffer (pH 7.6) containing LPS, MgSO_4_ (final concentration: 0.01 M), and Pb(NO_3_)_2_ (final concentration: 0.06% (wt/vol)) at 37 °C. Lead phosphate precipitates are formed at the site of enzyme activity, which are converted by incubation with Na_2_S to a lead sulphate, which appears as a dark brown staining. Specificity was checked by inhibition of AP activity using levamisole, an inhibitor of liver/bone/kidney AP [1]. In control incubations, the substrate LPS was omitted. Sections were counterstained with hematoxylin according to standard methods.

#### 2.6.3. Quantitative Analysis of Alkaline Phosphatase Activity in Tissue and Serum 

AP activity in the livers of mice was measured using QUANTI-Blue reagent (InvivoGen, Dan Diego, CA, USA) according to the manufacturer’s instructions. Tissue homogenates were diluted in RIPA/TRIS buffer to a protein concentration of 500 μg/mL. AP activity was expressed in mUnits per mg protein. AP activity in serum (U/L) was assayed at pH9.8 with para-nitrophenyl phosphate (pNPP, Sigma) as substrate according to standard procedures as previously described [8]. Serum samples of 5 μL were used for the measurement of AP activity.

### 2.7. Statistical Analysis

The results are expressed as the mean ± SEM, unless otherwise specified. Statistical analyses were performed using the Mann-Whitney U test (Graphpad Prism software). *p* < 0.05 was considered as the minimum level of significance.

## 3. Results

### 3.1. Effect of Lipid A Molecules on RAW 264.7 Macrophages

**Figure 1:**
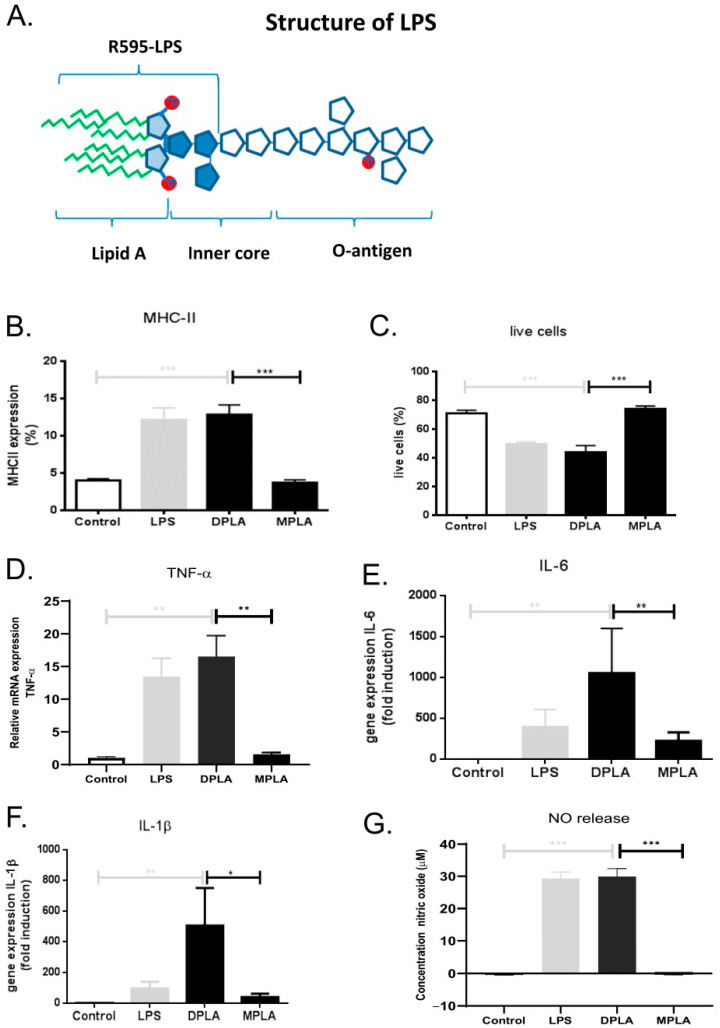
Schematic representation of the chemical structure of lipopolysaccharide (LPS) and the effects of full length LPS, di-phosphoryl lipid A (DPLA), and mono-phosphoryl Lipid A (MPLA) on RAW 264.7 cells in vitro. (**A**) General structure of wild-type LPS illustrating the lipid A moiety with two phosphate groups, the inner core and the O-antigen. Occasional phosphate groups may also be present in the highly variable O-antigen. The truncated form of LPS, i.e., DPLA, as used in the present study, is also indicated. (**B**,**C**) In vitro effects of LPS, DPLA, and MPLA on the expression of MHC class II and the percentage of live cells, as analysed by FACS, in cultures of RAW 264.7 cells. (**D**–**F**) In vitro effects of LPS, DPLA, and MPLA on the gene expression of TNFα, IL-1β, and IL-6, respectively, as analysed by qPCR in cultures of RAW264.7 cells. (**G**) Nitric oxide (NO) production of RAW264.7 cells 24 h after exposure to LPS, DPLA, or MPLA. The results show significant responses of macrophages to LPS, which serves as positive control, and DPLA, but no or limited response to MPLA. *n* = 5 per group (* = *p* < 0.05; ** = *p* < 0.01; *** = *p* < 0.001).

We first examined the effect of different lipid A molecules on the highly LPS-responsive macrophage cell line RAW 264.7. Wild-type LPS from *E. coli* served as positive control and the response of the R595 chemotypes for *Salmonella minnesota*, truncated to the lipid A moiety plus 3 KDO sugars of the inner core to maintain solubility (illustrated in Figure 1A), were tested. The truncated LPS chemotypes contain either two phosphate groups (di-phosphoryl lipid A or DPLA) or one phosphate group (mono-phosphoryl lipid A or MPLA). Both LPS and DPLA (final concentrations of 100 ng/mL) induced a strong increase in the expression of MHC class II in RAW cells (Figure 1B) relative to control incubations (addition of vehicle). The potency of LPS and DPLA was similar. LPS and DPLA also both induced a reduction in cell viability to a similar extent (Figure 1C). In contrast, de-phosphorylated lipid A (MPLA; 100 ng/mL) induced no MHC class II expression at all, nor did it affect cell viability in these macrophages (Figure 1B,C). In addition, mRNA expression levels for the pro-inflammatory mediators TNFα, IL-1β, and IL-6 were significantly enhanced after 24 h by LPS and DPLA, while MPLA did not induce the expression of any of these pro-inflammatory genes (Figure 1D–F). LPS and DLPA also induced significant NO production, while MPLA did not (Figure 1G). These observations confirm and extend our earlier studies on TNFα and NO responses by DPLA and MPLA in RAW 264.7 cells [9] and illustrate again that the lipid A moiety of LPS accommodates the pro-inflammatory activity of the LPS molecule where phosphate groups strongly determine its biological activities.

### 3.2. Effect of Lipid A Molecules on Primary Rat HSCs

**Figure 2:**
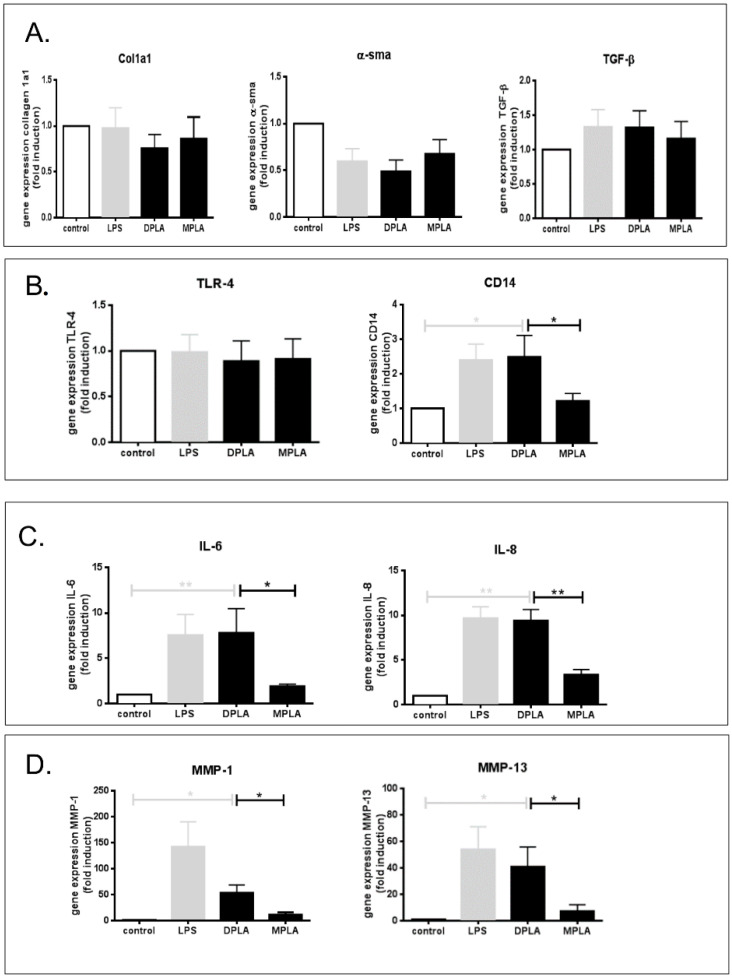
Effects of wild-type LPS, di-phosphoryl lipid A (DPLA), and mono-phosphoryl lipid A (MPLA) on gene expression levels in cultures of primary rat hepatic stellate cells (HSCs). (**A**) Effects of LPS, DPLA, and MPLA on mRNA levels of the fibrogenic parameters Collagen 1a1, αSMA, and TGFβ. (**B**) Effects of the bacterial toxins on mRNA levels of the LPS receptors TLR-4 and CD14. (**C**) Effects on mRNA levels of the proinflammatory mediators IL-6 and IL-8. (**D**) Effects of the bacterial toxins on mRNA levels of MMP-1 and MMP-13. LPS (from *E. coli*) serves as positive control (* = *p* < 0.05; ** = *p* < 0.01; *n* =5 per group).

As the profibrogenic activity of LPS has become clear in recent years, we tested the direct effects of different lipid A molecules on primary HSCs. In cultures of primary rat HSCs, LPS, DPLA, and MPLA did not directly affect *Col1a1* gene expression, nor TGFβ gene expression levels, and did not have a significant effect on αSMA mRNA levels (Figure 2A). Moreover, TLR-4 expression levels were not significantly altered by either compound (Figure 2B). However, mRNA levels for CD14, IL-6, and IL-8 were strongly enhanced by LPS and DPLA, whereas MPLA did not induce any significant effect on any of these parameters relative to control (Figure 2B,C). In parallel, MMP-1 and MMP-13 mRNA expression levels were significantly enhanced by LPS and DPLA, whereas MPLA had no significant effect on these parameters (Figure 2D). These data show that lipid A from *S. minnesota* has similar effects on HSC as full length LPS from *E. coli*, albeit the potency of DPLA seems higher than that of full length LPS, but MPLA, differing with DPLA on only one phosphate-position, has no effect at all on primary HSC.

### 3.3. Dephosphorylation of LPS and Lipid A in Mouse and Human Livers

**Figure 3:**
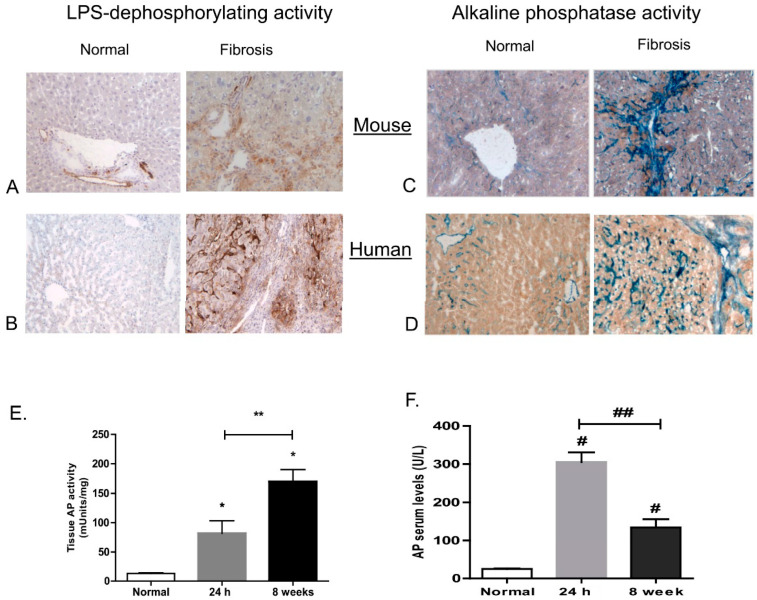
LPS-dephosphorylating (**A**,**B**) and alkaline phosphatase (AP) activity (**C**,**D**) in normal and fibrotic livers of mouse and man. Representative pictures of LPS-dephosphorylating activity in mouse (**A**) and human liver tissue (**B**). Brown staining represents sites of enzymatic phosphate release at pH 7.6 LPS from *E. coli* as substrate (blue is haematoxylin). (**C**,**D**) AP activity in mouse and human livers, respectively. Blue staining represents phosphatase activity using the substrate Naphthol-ASMX-phosphate at pH 9.8. (**E**,**F**) Quantification of AP activity in liver tissue (**E**) and in serum (**F**), at t = 24 h and t = 8 weeks of the CCl_4_ administration protocol. Note the LPS-dephosphorylating activity in hepatocytes and hepatic arteries and the increase in fibrotic livers relative to normal, in both mouse and human tissue. Abbreviations: ha = hepatic arterioles, bd = bile duct, pv = portal vein, hep = hepatocytes. * = *p* < 0.05 compared with normal, ** = *p* < 0.05 24 h vs. 8 weeks CCl_4_, # = *p* < 0.01 compared with normal. ## *p* < 0.01 24 h vs. 8 weeks CCl_4_.

We subsequently investigated whether hepatic LPS-dephosphorylating activity can be detected in situ. In normal mice, histochemical staining showed clear enzymatic phosphate release upon incubation of liver sections with *E. coli* LPS around hepatic arteries and arterioles (Figure 3A). In fibrotic livers, a clear increase in LPS-dephosphorylating activity was seen compared with normal livers. In particular, in fibrotic areas, significant LPS-dephosphorylating activity was detected in the cytoplasm of hepatocytes (Figure 3A). In contrast, no activity was found upon incubation without substrate (not shown). The specificity of this staining has previously been demonstrated by addition of levamisole, an inhibitor of liver/bone/kidney AP activity [2].

Staining for AP activity according to conventional methods using Naphthol AS-MX-phosphate as substrate revealed high enzyme activity (blue staining) at similar localizations as LPS-dephosphorylating activity (Figure 3C).

LPS-dephosphorylating activity was also found in human livers; while in normal human livers, activity was very low, high LPS dephosphorylating activity was found in cirrhotic livers (Figure 3B). This activity was most prominent along hepatocytes in their canalicular and basolateral membranes and in hepatic arterioles, similar to the localisation described for alkaline phosphatase activity in normal and cirrhotic human livers [39]. Staining for AP activity using Naphthol AS-MX-phosphate also revealed a strong staining in fibrotic livers relative to normal livers. The AP staining pattern corresponded with LPS-dephosphorylating activity, i.e., along arteries, hepatocytes, and fibrotic bands (Figure 3D). In mice, intrahepatic and serum AP levels were already significantly elevated 24 h after the first injection of CCl_4_ (Figure 3E,F). After 8 weeks, the time point corresponding to the histochemical pictures, intrahepatic AP levels were further increased, whereas serum AP levels were significantly declined relative to the situation at 24 h.

In summary, LPS-dephosphorylating activity was found in normal mouse and human livers, which was increased in fibrotic livers of both species. Staining corresponded with the localization of liver/bone/kidney AP activity in these livers. Previous studies have already shown high LPS-dephosphorylating activity within the intestinal wall [38].

### 3.4. Effect of Exogenous iAP on Liver Fibrogenesis

**Figure 4:**
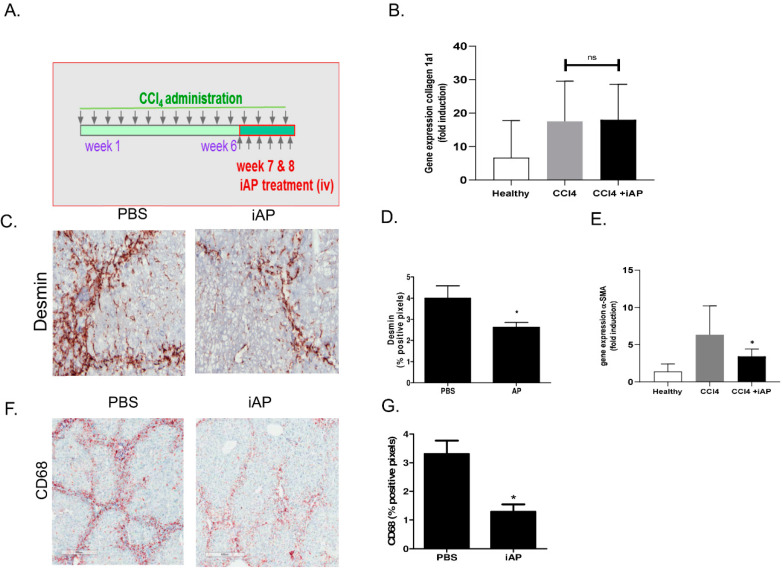
Effect of exogenously administered iAP on CCl_4_-induced liver fibrosis. (**A**) Scheme depicting the administration schedule of CCl4 (i.p.) to Balb/c mice and the subsequent treatment regimen with iAP (i.v) in the final 2 weeks. (**B**) Collagen 1a1 gene expression levels in healthy mice and fibrotic animals with and without treatment. (**C**) Representative pictures of desmin staining in livers of fibrotic mice treated with Phosphase-buffered saline (PBS) or iAP. (**D**) Quantification of hepatic desmin staining in fibrotic mice treated with PBS or iAP using Cell D image analysing software. (**E**) α-SMA gene expression levels in healthy mice and fibrotic animals with and without treatment. (**F**) Representative pictures of CD68 staining in livers of fibrotic mice treated with PBS or iAP. (**G**) Quantification of intrahepatic CD68 staining in fibrotic mice treated with PBS or iAP using Cell D image analysing software. Magnification of sections: 100×. Bars represent mean ± SEM of 5–6 mice per group (ns = not significant, * = *p* < 0.05).

We subsequently tested the role of AP by treating mice with CCl_4_-induced liver fibrosis for 2 weeks with iAP (i.v. three times a week in week 7 and 8, see scheme in Figure 4A), with the aim to dephosphorylate circulating LPS. The CCl_4_ model is characterized by translocation of LPS into the systemic circulation, which stimulates the progression of fibrosis [34,35,36]. In the studied time-frame, significant collagen deposition occurs, but *col1a1* gene expression was not attenuated by iAP treatment (Figure 4B). However, the number of desmin-positive cells in CCl4-treated mice was clearly reduced after iAP treatment (Figure 4C). Moreover, αSMA mRNA expression levels in livers of iAP-treated mice were reduced compared with untreated fibrotic mice (*p* < 0.05, Figure 4D). Staining for CD68-positive macrophages was also reduced in iAP-treated mice compared with untreated fibrotic mice (Figure 4E,F). Further analysis of the intrahepatic activities of these cells yielded inconclusive results. In conclusion, iAP-treatment of CCl_4_-mice reduced the number of macrophages in the liver, as well as the number of desmin-positive and αSMA positive-fibroblasts, with no detectable change in collagen deposition.

## 4. Discussion

The present study shows that LPS, in particular the lipid A moiety thereof, significantly affects macrophage and fibroblast activities, and thus the fibrogenic process within the fibrotic liver.

A relatively small part of LPS, the lipid A moiety, harbours most of the biological activities of the whole molecule [3]. Although there is some variability in the fatty acid chains of this lipid A moiety, the general structure of lipid A is rather constant among most bacterial species [3]. We and others have shown that the two phosphate groups in this lipid A moiety are very important for its biological activities [3,4,5,6,9].

During chronic liver diseases, gut-derived LPS is known to promote fibrogenic activity [25,40,41]. Because of increased permeability of the intestinal wall, associated with high levels of permeability-enhancing factors in the circulation, increased levels of LPS and other bacterial products enter the portal circulation [42]. In a NASH animal model, externally-induced colitis led to higher portal LPS levels, which was followed by hepatic inflammation and fibrogenesis [43]. Many other studies have shown increased translocation of LPS and bacteria during liver fibrosis in patients and in several animal models of fibrosis such as the high fat-diet [29] and the CCl_4_ model [34,35,36]. Subsequently, liver damage leads to an increased expression of LPS-responsive receptors [18,24,25,29]. In the CCl_4_ model, not only does CCl_4_ induce liver damage, but also the repeated i.p. administration of olive oil for 8 weeks may affect hepatocyte physiology, as reflected by the occasional visibility of lipid-filled vacuoles in these cells (not shown). For that reason, we did not administer olive oil to the control group to ensure the presence of completely normal livers in this group. In normal livers, TLR4 expression by resident hepatic cells is low, but during liver fibrogenesis, TLR4 expression is strongly enhanced [18]. Thus, the increased delivery of LPS to the liver is paralleled by an increased responsiveness of many liver cells to this molecule [29], thus creating a profibrotic positive feedback loop.

To examine the relevance of phosphate groups in the lipid A moiety of LPS on this profibrotic feedback loop, we examined the effects of mono- and di-phosphoryl lipid A on macrophages and hepatic stellate cells. Wild-type LPS is a highly variable structure and may, next to the two phosphate groups in the toxic lipid A moiety, also have some phosphate groups in the long polysaccharide tail with little or less biological activity [3]. This would complicate our conclusions, so we applied well-defined lipid A molecules. In the R595-chemotypes we used, the long polysaccharide tail is not present, except three 2-keto-3-deoxymanno-octulosonic acid (KDO) sugars of the inner core (illustrated in Figure 1A) to circumvent insolubility of the lipid A part. The only difference between mono- and di-phosphoryl lipid A is one phosphate group. Lipid A contains two glucosamine sugars, generally both substituted with a phosphate group. Naturally occurring MPLA, produced by some bacterial strains [44], can either lack the phosphate group at the 1 position or at the 4′position, and commercially available MPLA preparations are a mixture of both types of MPLA. To date, it is unknown which phosphate group is removed by AP. Using histochemical methods, we have not seen dephosphorylation of MPLA (results not shown), so apparently not both. Our results show that the removal of one phosphate group profoundly alters the pro-inflammatory and fibrogenic activities. Wild-type LPS and di-phosphoryl lipid A both strongly activated RAW macrophages and primary isolated HSCs, as reflected by an enhanced expression of MHC II by RAW cells and several pro-inflammatory cytokines by both cell types (Figure 1 and Figure 2). In RAW cells, DPLA was even more potent than the whole LPS molecule, which might be related to a difference in bacterial source (*E. coli* versus *S. minnesota*). In HSC, LPS and DPLA displayed similar activities (except on MMP-1 gene expression), despite their large structural differences, confirming that the lipid A moiety is essential for many biological activities of LPS. However, mono-phosphoryl lipid A induced no such activity at all in both cell types. MPLA did not induce inflammatory or fibrotic parameters, nor in the highly responsive RAW macrophages, nor in HSCs. This is in agreement with our earlier studies showing no TNFα and NO production of macrophages stimulated with MPLA, in contrast to LPS and DPLA [9]. It should be noted that, in our studies, LPS and DPLA did not stimulate collagen production, αSMA expression, or TGBβ-expression directly in primary HSCs, so the profibrotic activities of this compound in vivo may be indirect through immunostimulatory processes via macrophages or via pro-inflammatory cytokines like IL6 and IL8. Interestingly, LPS and DPLA directly stimulate the expression of LPS-responsive elements in HSC, thus creating a positive feedback loop, whereas MPLA does not. It can thus be concluded that the removal of one phosphate group from the lipid A moiety of LPS greatly affects the responses of the key effector cells of fibrosis on this bioactive molecule.

LPS-dephosphorylating activity in several tissues, including rat livers, has been demonstrated by us [1,9,38], as well as its upregulation in bile duct ligated rats [45]. We now examined whether this activity is also increased in CCL_4_-induced liver fibrosis in mice and in human cirrhotic patients. The present study shows LPS dephosphorylation in normal mouse and human livers, mostly along arteries, and a significant upregulation of this LPS-dephosphorylating activity in fibrotic livers of mice and cirrhotic patients. This activity was found to be co-localized with AP activity in all species tested. In particular, in the fibrotic areas around activated HSC, high LPS-dephosphorylating activity was found. Our results showed a significant and gradual increase in intrahepatic AP activity during liver fibrogenesis, which did not correlate with serum AP activity, which was also increased during fibrosis, but declined after an initial elevation in the acute phase. Apparently, both increments are differently regulated.

Intestinal AP is a brush border enzyme expressed as an ecto-enzyme in the intestinal lumen [1]. It was already described in 1934, but, because of its extremely high pH optimum (above 10), which is incompatible with life, the physiological relevance of AP has remained unclear. We found that AP is able to dephosphorylate and detoxify LPS at pH 7.5 [1,2]. Since then, the role of AP has been explored in several LPS-induced diseases, most notably in clinical Phase II trials in patients with inflammatory bowel diseases and in patients with acute renal failure [13,14,15,17].

We hypothesized that iAP may also protect the liver against LPS-mediated effects. iAP is taken up by the asialoglycoprotein receptor [46] expressed on hepatocytes, and high delivery of this isoenzyme to the liver after its systemic administration has already been demonstrated [45]. There are four isoenzymes in the body: intestinal AP, liver-bone-kidney AP (or tissue-non-specific AP), placental AP, and Hodgekin lymphoma-associated AP [47]. It is unknown which iso-enyzme is most the potent to dephosphorylate LPS. Histochemical studies show the strongest LPS-dephosphorylating activity in the intestine [2], but whether this is because of a higher potency of this isoenzyme or higher protein expression at this site is unknown.

To test whether this intestinal-AP is able to attenuate liver fibrogenesis, we administered exogenous intestinal AP into the circulation of mice with CCL_4_-induced liver fibrosis. Systemic administration of iAP did not result in a significant reduction of collagen deposition. However, several parameters reflecting HSC accumulation or activation (desmin, aSMA) were significantly reduced and significant effects were also seen on parameters reflecting macrophage accumulation (CD68). The mechanism of action of iAP in vivo cannot be explored in detail because dephosphorylated LPS cannot be detected in vivo, but the CCL_4_-model of fibrogenesis is associated with increased circulating levels of LPS [34,35,36], and the significance of phosphate groups of lipid A has been shown in vitro here. The effects of iAP on liver fibrogenesis are in line with our hypothesis that circulating AP is able to dephosphorylate circulating LPS. Intestinal AP is released in the circulation [46,47,48] and its rapid uptake by the liver [45] may suggest that iAP is an intrinsic part of the liver–gut axis. Elevated intestinal AP levels in the circulation protected mice from sepsis [46]. Other studies have shown that iAP also affects the mibrobiome [32,49,50], possibly by interfering with the ATP content in the intestinal lumen [51], and the microbiome in turn affects liver physiology [52]. Intestinal AP is influenced by enteral nutrition and it can restore gut microbiota and reduce pathogen colonization [53,54]. IAP administration prevents the metabolic syndrome in a mouse model [55], possibly by detoxification of intraluminal LPS, thus preventing translocation of gut-derived LPS and the subsequent low-grade inflammation that triggers the metabolic syndrome [55], and even affects aging [56]. In addition, in a recent study, it was found that intestinal AP activity is reduced in patients and mice with liver fibrosis, and that the fibrogenic process within the liver can be inhibited by restoration of the intestinal activity via oral administration of iAP [57].

Obviously, intestinal concentrations of di-phosphoryl lipid A are high and, in several diseases, increased levels of this bacterial product may leak from the gut into the circulation, which may lead to sepsis and or tissue damage in other organs. The fact that AP is able to dephosphorylate LPS may thus represent an important first line of defence against this very bioactive molecule, with several studies lending support to this hypothesis [12,46]. Following successful Phase II trials in patients with acute kidney injury associated with sepsis [17] and inflammatory bowel diseases [14], a multi-national Phase III trial of recombinant AP in 1400 patients with sepsis-associated-acute kidney injury has recently been initiated. The present study support and extends this, defining AP as a protective enzyme with regulatory effects on pro-fibrogenic processes within the liver.

In conclusion, the present study shows that phosphate groups in the lipid A moiety of LPS strongly affect the response of HSCs and macrophages on this bacterial product. LPS dephosphorylation by intestinal [38] and hepatic phosphatases, as demonstrated histochemically here, may thus represent a protective barrier to protect the liver from gut-derived LPS. The study sheds new light on a physiological role for AP, which may have a pathophysiological role in liver diseases, next to its role as a marker.

## Figures and Tables

**Table 1:** Primers used. TLR, Toll-like receptor.

Mouse primers:		
**Gene**	**Forward Primer**	**Reverse Primer**
β-actin	5′ ATC GTG CGT GAC ATC AAA GA 3′	3′ ATG CCA CAG GAT TCC ATA CC 5′
TNF-α	5′ CAT CTT CTCA AAA TTC GAG TGA CAA 3′	3′ GAG TAG ACA AGG TAC AAC CC 5′
IL-1β	5′ GCC AAG ACA GGT CGC TCA GGG 3′	3′ CCC CCA CAC GTT GAC AGC TAG G 5′
IL-6	5′ TGA TGC TGG TGA CAA CCA CGG C 3′	3′ TAA GCC TCC GAC TTG TGA AGT GGT A 5′
Col1a1	5′ TGA CTG GAA GAG CGG AGA GT 3′	3′ ATC CAT CGG TCA TGC TCT CT 5′
αSMA	5′ ACT ACT GCC GAG CGT GAG AT 3′	3′ CCA ATG AAA GAT GGC TGG AA 5′
Rat primers:		
**Gene**	**Forward Primer**	**Reverse Primer**
β-actin	5′ GGC ATC CTG ACC CTG AAG TA 3′	5′ GGG GTG TTG AAG GTC TCA AA 3′
IL-6	5′ CCG GAG AGG AGA CTT CAC AG 3′	5 ‘ACA GTG CAT CAT CGC TGT TC 3′
IL-8	5′ GGC AGG GAT TCA CTT CAA GA 3′	5′ GCC ATC GGT GCA ATC TAT CT 3′
TLR4	5′ AAC TTC CTG GGG AAA AAC TCT TG 3′	5′ TGC CAC CAT TTA CAG TTC GTC AT 3′
CD14	5′ GTT ACA CAA CAG GCT GGA TAG G 3′	5′ ACT ACG CCA GAG TTA TAC GC 3′
MMP-1	5′ CTT GCG GGA ATC CTG AAG AAG TCT A 3′	5′ GCC AAG CTC ATG GGC AGC AAC AAT 3′
MMP-13	5′ GGA AGA CCC TCT TCT TCT CA 3′	5′ TCA TAG ACA GCA TCT ACT TTG TC 3′
TGFβ	5′ ATA CGC CTG AGT GGC TGT CT 3′	5′ TGG GAC TGA TCC CAT TGA TT 3′
Col1a1	5′ AGC CTG AGC CAG CAG ATT GA 3′	5′ CCA GGT TGC AGC CTT GGT TA 3′
αSMA	5′ GAC ACC AGG GAG TGA TGG TT 3′	5′ GTT AGC AAG GTC GGA TGC TC 3′

## Supplementary Materials

The following are available online at https://www.mdpi.com/2073-4409/9/12/2708/s1. Supplementary Table S1. Patient characteristics of the human livers used.
