# Supplementary material for: Phosphate Groups in the Lipid A Moiety Determine the Effects of LPS on Hepatic Stellate Cells: A Role for LPS-Dephosphorylating Activity in Liver Fibrosis
Source: Cells. 2020 Dec 17;9(12):2708. doi: 10.3390/cells9122708 (PMC7766276; doi:10.3390/cells9122708)
Supplement: Supplementary file 1 [file cells-09-02708-s001.pdf]

Supplementary Table

Table 1: Patient characteristics of the human livers used.

	Normal livers	Cirrhotic livers
	N=7	N=6
Age (min-max) years	41 (10-57)	49 (35-66)
Gender	n=4: F<br>n=2: M<br>n=1: not known	n=3: F<br>n=1: M<br>n=2: not known
